# *Plasmodium vivax* malaria incidence over time and its association with temperature and rainfall in four counties of Yunnan Province, China

**DOI:** 10.1186/1475-2875-12-452

**Published:** 2013-12-18

**Authors:** Nicola A Wardrop, Adrian G Barnett, Jo-An Atkinson, Archie CA Clements

**Affiliations:** 1 University of Southampton, Geography and Environment, Highfield Campus, University Road, Southampton SO17 1BJ, UK; 2 Queensland University of Technology, School of Public Health and Social Work, Victoria Park Road, Kelvin Grove, Queensland 4059, Australia; 3 Infectious Disease Epidemiology Unit, University of Queensland, School of Population Health, Public Health Building, Herston Road, Herston, Queensland 4006, Australia

**Keywords:** Distributed lag non-linear model, Generalized linear Poisson model, *Plasmodium vivax* malaria, Time series analysis, Weather variables

## Abstract

**Background:**

Transmission of *Plasmodium vivax* malaria is dependent on vector availability, biting rates and parasite development. In turn, each of these is influenced by climatic conditions. Correlations have previously been detected between seasonal rainfall, temperature and malaria incidence patterns in various settings. An understanding of seasonal patterns of malaria, and their weather drivers, can provide vital information for control and elimination activities. This research aimed to describe temporal patterns in malaria, rainfall and temperature, and to examine the relationships between these variables within four counties of Yunnan Province, China.

**Methods:**

*Plasmodium vivax* malaria surveillance data (1991–2006), and average monthly temperature and rainfall were acquired. Seasonal trend decomposition was used to examine secular trends and seasonal patterns in malaria. Distributed lag non-linear models were used to estimate the weather drivers of malaria seasonality, including the lag periods between weather conditions and malaria incidence.

**Results:**

There was a declining trend in malaria incidence in all four counties. Increasing temperature resulted in increased malaria risk in all four areas and increasing rainfall resulted in increased malaria risk in one area and decreased malaria risk in one area. The lag times for these associations varied between areas.

**Conclusions:**

The differences detected between the four counties highlight the need for local understanding of seasonal patterns of malaria and its climatic drivers.

## Background

In the past, *Plasmodium vivax* malaria has been considered to be a less severe form of malaria in comparison to *Plasmodium falciparum*, contributing to continuing gaps in our understanding of *P. vivax* malaria epidemiology [1]. However, this view has been criticized in light of evidence that *P. vivax* can contribute significant levels of morbidity and mortality in some areas [2,3]. Within China, the majority of malaria cases are caused by *P. vivax*, with transmission occurring predominantly in central and southern areas. Yunnan and Hainan provinces in the country’s south experience the highest incidence rates [4], although large reductions in malaria incidence have been observed across China in the past few decades, concurrent with global decreases in incidence [5-8]. Malaria elimination is now a priority for the World Health Organization, and within China the focus of malaria activities has recently been refocused towards malaria elimination by 2020 [9].

Transmission of malaria depends on: the availability of competent vectors, parasite development, vector biting rates and other factors. Environmental factors such as temperature and rainfall have been shown to strongly influence malaria transmission and differential seasonal patterns across areas. Water (and therefore, rainfall) is necessary for mosquito breeding, the development of mosquito larva and adult mosquito numbers [10]. However, excessive rainfall volumes may reduce numbers due to flood waters washing away *Anopheles* larvae [11,12]. Temperature influences both mosquito populations and the *Plasmodium* parasites, as the speed of both larval development and parasite maturation increases at higher temperatures [12,13], although this response is non-linear with detrimental effects on mosquito survival above a threshold temperature. The frequency of mosquito feeding also increases with temperature, which has additional impacts on malaria transmission dynamics [14]. These climatic dependencies drive the latitudinal and altitudinal limits on the spatial distribution of malaria, and also determine seasonal patterns of malaria incidence.

Despite the number of publications demonstrating an association between malaria seasonality and temperature and rainfall, it is clear that these associations are not consistent across different areas, preventing the generalization of results [12]. The responses of mosquitoes to climate and rainfall can vary between locations, and the factors that drive this are poorly understood [15]. Improved understanding of these local relationships within endemic areas would provide significant evidence for the modification of malaria control and elimination programmes: for example, timing of interventions such as insecticide-treated bed net (ITN) delivery, or indoor residual spraying. Here, a *P. vivax* time-series from four counties of Yunnan province, southwest China was reported and analysed using seasonal trend decomposition and distributed lag non-linear models. The research aimed to (a) describe the seasonal patterns and temporal trends in malaria incidence, (b) examine the potential role of anomalous weather events (i.e. beyond the normal seasonal variability) on atypical malaria incidence and (c) examine the delayed effects of rainfall and temperature on malaria incidence, for all four of the study counties.

## Methods

### Study area

**Figure 1:**
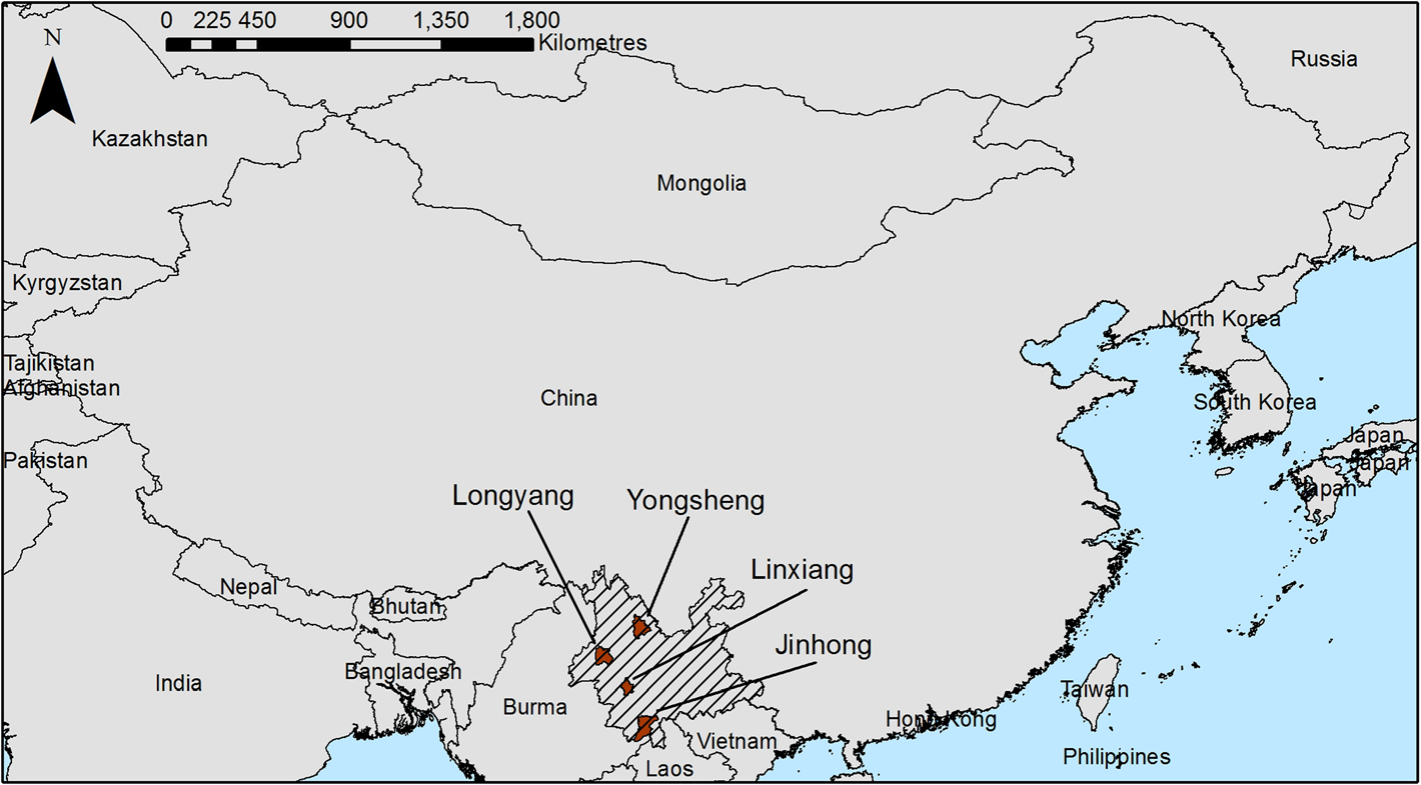
Map of China, highlighting Yunnan Province (hatched) and the four counties (brown).

Yunnan Province is located in the south of China (Figure 1). Four counties distributed across Yunnan Province were selected for analysis (note that the term county is used here to describe third-level administrative areas that are denoted as counties, districts or metropolitan areas), based on a high recorded incidence of malaria and availability of meteorological data. These were Jinhong, which is in the south of Yunnan province, Longyang in the west of Yunnan, Yongsheng in the north of Yunnan, and Linxiang in the southwest of Yunnan.

### Epidemiological data

*Plasmodium vivax* malaria surveillance data, aggregated to months, were obtained from the Yunnan Institute for Parasitic Diseases. Malaria is a notifiable disease in China, therefore, clinical malaria is subject to routine surveillance nationwide. Malaria surveillance in China is generally based on clinical symptoms and laboratory diagnosis; approximately 82% of cases in 2011 were laboratory confirmed, although this proportion may be higher than during the study period due to malaria elimination efforts, which were launched in July 2010 [16,17]. This study used these surveillance data from January 1991 to December 2006 for the four counties, during which period annual case counts ranged from 394 to 675 for Jinhong; 67 to 133 for Longyang; 35 to 105 for Yongsheng; and 34 to 55 for Linxiang. The data used were anonymised secondary data from a pre-existing source: in accordance with University of Southampton Ethics Committee guidance, no ethical approval was necessary.

### Weather and demographic data

Average monthly temperature and rainfall in each of the four counties was obtained from the National Meteorological Information Centre of China for January 1991 to December 2006. These data were collected from weather stations located within the counties. Population data were obtained from the 1990 and 2000 national censuses: these were used to calculate a linear monthly population growth rate, which was used to estimate monthly population counts.

### Exploration of seasonal patterns and temporal trends

The average monthly malaria incidence, rainfall and temperature were calculated from the full time-series for each of the four counties. These were plotted to show the average seasonal patterns in malaria and weather variables. For each county, the time series of malaria incidence was decomposed using seasonal-trend decomposition based on locally weighted regression [18] to show: the seasonal pattern, the temporal trend and the residual variability. This method uses loess smoothing on sub-series of each season (i.e. month in this case) separately to estimate the seasonal pattern. The seasonal component is then removed from the time series prior to further smoothing to estimate the trend, leaving the residual values.

### Correlation between atypical weather and malaria incidence variability

The typical seasonal patterns were removed from the temperature and rainfall data using a linear regression with month as an explanatory variable. A temporal trend was also included as an explanatory variable (a continuous variable derived from month and year), where this resulted in a decrease in model deviance and Akaike Information Criterion (AIC). The residual variation in rainfall and temperature was plotted over time and compared with the residual variability in malaria incidence from the decomposed time-series. Correlations were calculated to examine the relationship between atypical weather (following the removal of seasonal effects) and variability in malaria incidence.

### Distributed lag non-linear Poisson regression analysis

Poisson regression with distributed lag non-linear models was used to explore associations between incidence of *P. vivax* malaria and temperature and rainfall. An offset of monthly population and days of the month was used to control for changes in population size and the number of days in each month. For each county, the model that had the lowest AIC was selected. Month was included as an indicator variable for Jinhong and Longyang to account for seasonality. Due to parameter estimation instability, an indicator variable for season (seasons ran from December-February; March-May; June-August; September-November) was included in the model for Yongsheng rather than month, but neither was included in the model for Linxiang. Long-term trend was accounted for by including date (month and year) as a continuous variable. The trend was modelled as linear and non-linear (with two or three degrees of freedom (*df*)) during model comparison. Average monthly rainfall and temperature were included at varying temporal lags (zero to three months) using cross-basis matrices, created using the R package “dlnm” [19]. Three months was chosen as the maximum biologically plausible lag between malaria incidence and temperature or rainfall. Again, these relationships were included as both linear and non-linear effects, and log-transformations of the weather variables were also considered during model comparison. Contour plots of the relative risk were used to show the effect of temperature and rainfall on malaria incidence at different lags. The estimated trends were also plotted. All analyses were conducted using the R software [20].

## Results

### Exploration of seasonal patterns and temporal trends

**Figure 2:**
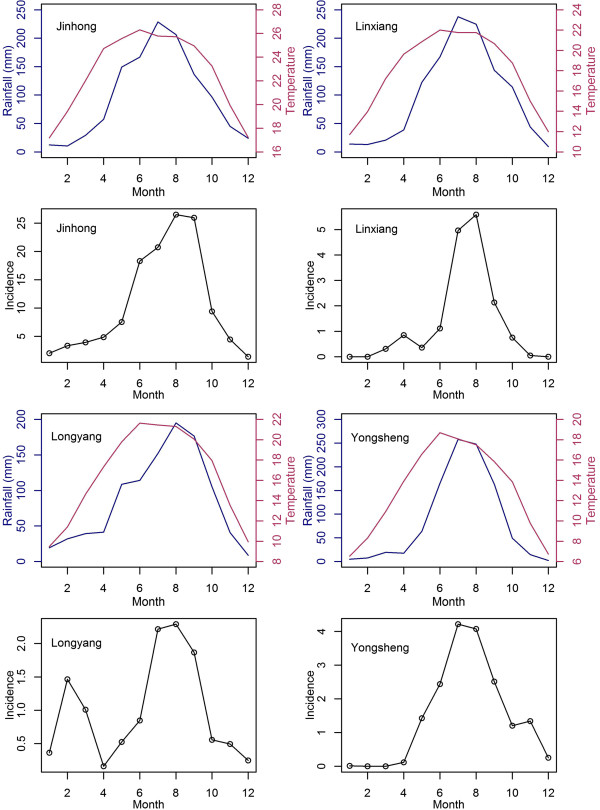
Monthly averages of rainfall (blue), temperature (red) and incidence rates per 100,000 population (black) from Jinhong, Linxiang, Longyang and Yongsheng.

The seasonal patterns of rainfall, temperature and malaria incidence in each of the four counties are in Figure 2. All four counties have warm summers with high rainfall and cooler winters with smaller rainfall volumes. Jinhong (elevation approximately 560 m) has the warmest climate, followed by Linxiang (elevation approximately 1,517 m), Longyang (elevation approximately 1,680 m), and Yongsheng (elevation approximately 2,400 m). Summaries of the monthly average malaria incidence and weather variables are in Table 1. In all four counties, peak malaria incidence was between July and September, corresponding with peak rainfall during the summer season. Additional, smaller peaks in malaria were seen for both Longyang and Linxiang, which coincide with higher monthly rainfalls during January and February. Overall incidence was substantially higher in Jinhong than the other three areas.

**Table 1:** Summary statistics for the average monthly malaria incidence and weather variables

**Area**	**Rainfall**	**Temperature**	**Malaria incidence per 100,000 population**
	**Average (min-max)**	**Average (min-max)**	**Average (min-max)**
**Jinhong**	97 mm	22 · 7°C	10 · 7
	(10 · 4-228 · 4 mm)	(17 · 2-26 · 3°C)	(1 · 4-26 · 5)
**Linxiang**	96 mm	17 · 9°C	1 · 3
	(9 · 3-237 · 8 mm)	(11 · 7-22 · 0°C)	(0-5 · 6)
**Longyang**	86 · 1 mm	16 · 5°C	1
	(8 · 8-195 · 1 mm)	(9 · 5-21 · 6°C)	(0 · 2-2 · 3)
**Yongsheng**	84 · 4 mm	13 · 1°C	1 · 5
	(2 · 3-257 · 9 mm)	(6 · 5-18 · 7°C)	(0-4 · 2)

**Figure 3:**
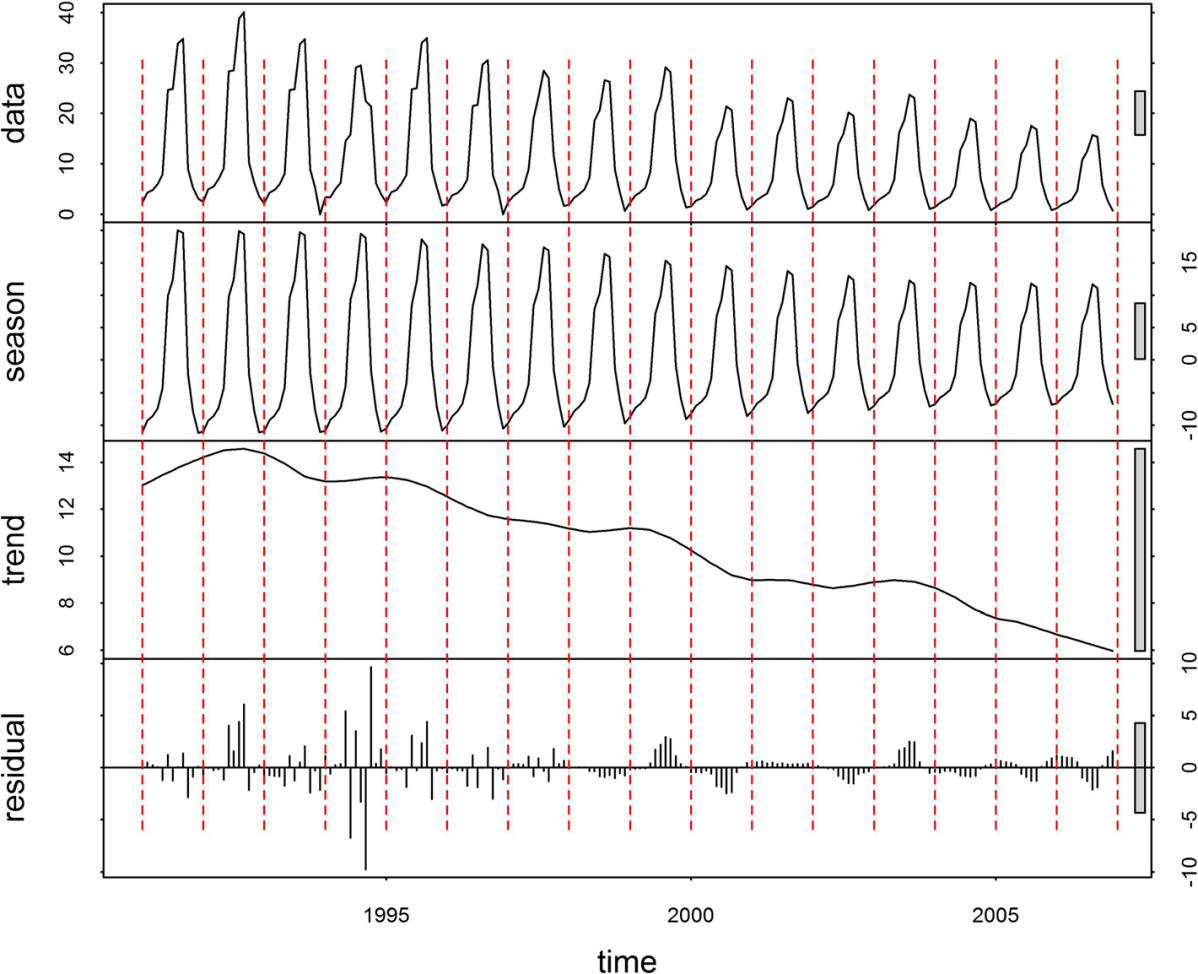
**Decomposed *Plasmodium vivax *time-series for Jinhong.** Data presented as incidence rates per 100,000 population.

**Figure 4:**
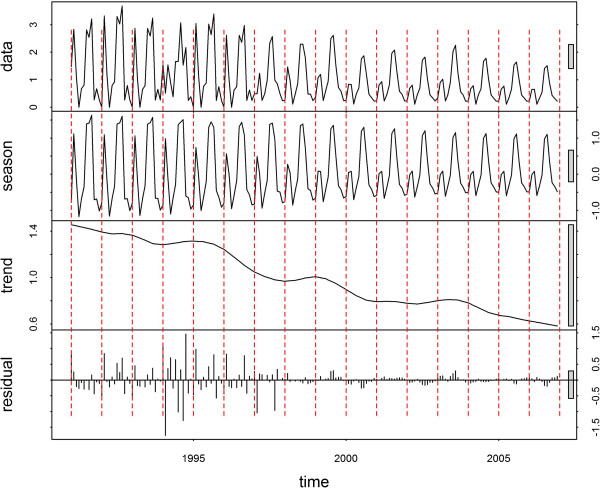
**Decomposed *Plasmodium vivax *time-series for Longyang.** Data presented as incidence rates per 100,000 population.

**Figure 5:**
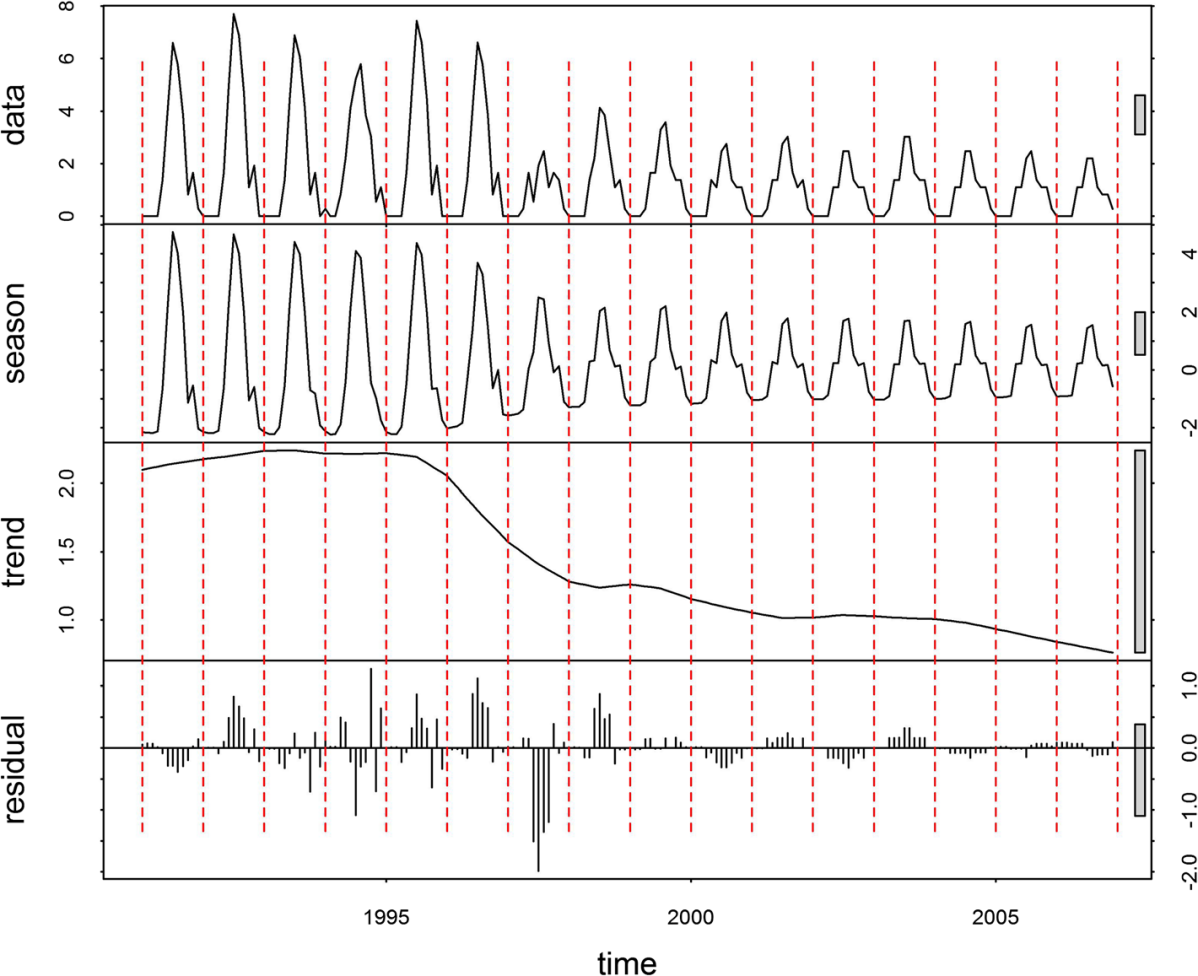
**Decomposed *Plasmodium vivax *time-series for Yongsheng.** Data presented as incidence rates per 100,000 population.

**Figure 6:**
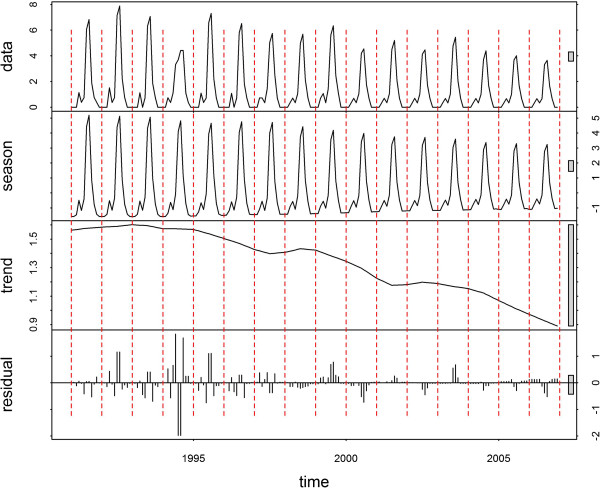
**Decomposed *Plasmodium vivax *time-series for Linxiang.** Data presented as incidence rates per 100,000 population.

The time-series decompositions are shown for Jinhong, Longyang, Yongsheng and Linxiang (Figures 3, 4, 5 and 6, respectively). A clear seasonal pattern is evident in the raw data (top section) in all four counties. Jinhong and Yongsheng had single annual peaks of incidence, whereas Longyang and Linxiang had a main peak in the summer with a smaller peak earlier in the year. For all four counties, the amplitude of the seasonal pattern decreased over time due to decreasing incidence. The residual variation (bottom section) for all four counties showed two distinct patterns, with smaller temporally correlated residuals occurring after approximately 1998 or 1999, in comparison with larger and more random residual variation before this time.

### Correlation between atypical weather and malaria incidence variability

A comparison of the residual variation in malaria incidence from the time-series decomposition and residual variation in temperature and rainfall (following removal of the seasonal pattern using linear regression) did not highlight any correlation between seasonal anomalies in weather and malaria incidence (see Additional file 1: Table S1).

### Distributed lag non-linear Poisson regression analysis

**Figure 7:**
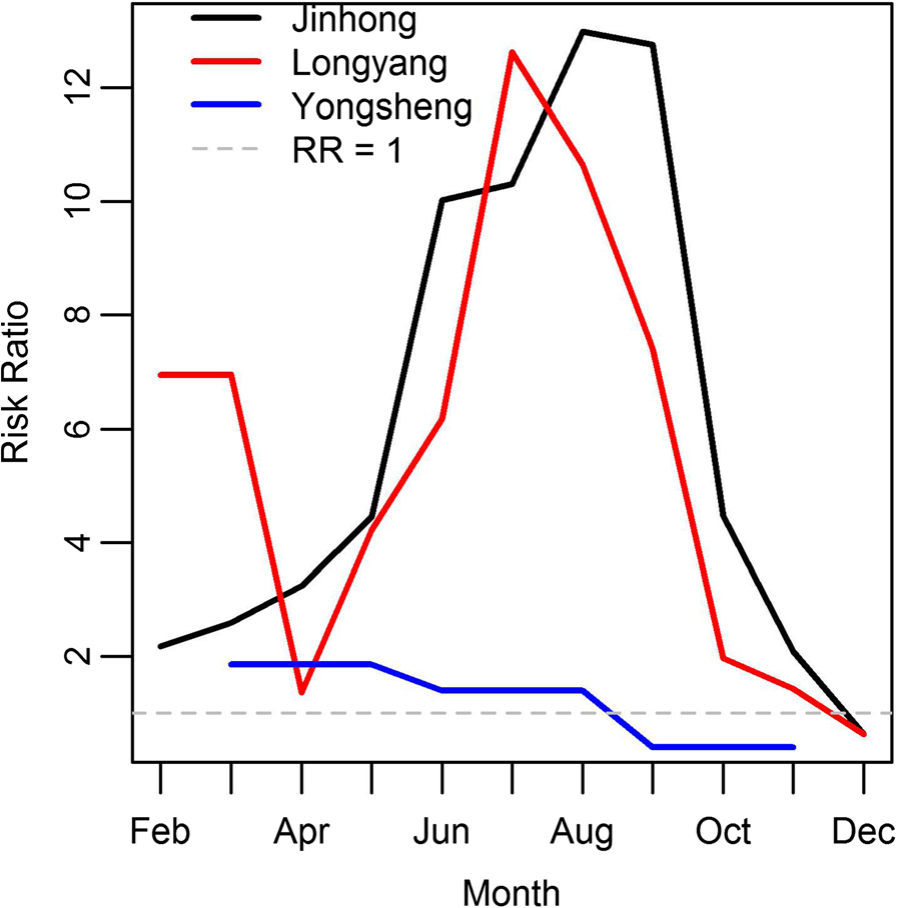
**Plot of risk ratios of malaria incidence by months (or seasons for Yongsheng).** *Neither month nor season were included in the model for Linxiang due to parameter estimation instability, hence, no risk ratios are presented for Linxiang.

The regression models are presented in Additional file 2: Table S2, Additional file 3: Table S3, Additional file 4: Table S4, Additional file 5: Table S5. Linear trends were used for Longyang and Yongsheng, and natural cubic splines, with two *df*, were used to model the trend in Jinhong and Linxiang (see Additional file 6: Figure S1). For all four areas, the relative risk of malaria decreased over time. In Jinhong, Longyang and Yongsheng, the relative risk decreased steadily, whereas in Linxiang the relative risk was static until 1997, after which it decreased (although confidence intervals for the initial four years are wide). The estimated risk ratio (RR) for each month (or season in the case of Yongsheng) is in Figure 7 (the reference categories were January for Jinhong and Longyang and winter (December to February) for Yongsheng). In Jinhong, the lowest RR was in December, with all other months demonstrating increased risk of malaria in comparison with the reference month (January), and a peak RR of 13 during August. A slightly different pattern is evident for Longyang. Again, the lowest RR was in December, with RRs greater than one during the summer months, peaking at 12.6 in July. However, a smaller peak also occurred in February and March, with RRs of 7. For Yongsheng, RRs were greater than one during spring (March to May) and summer (June to August), and less than one during autumn (September to November).

**Figure 8:**
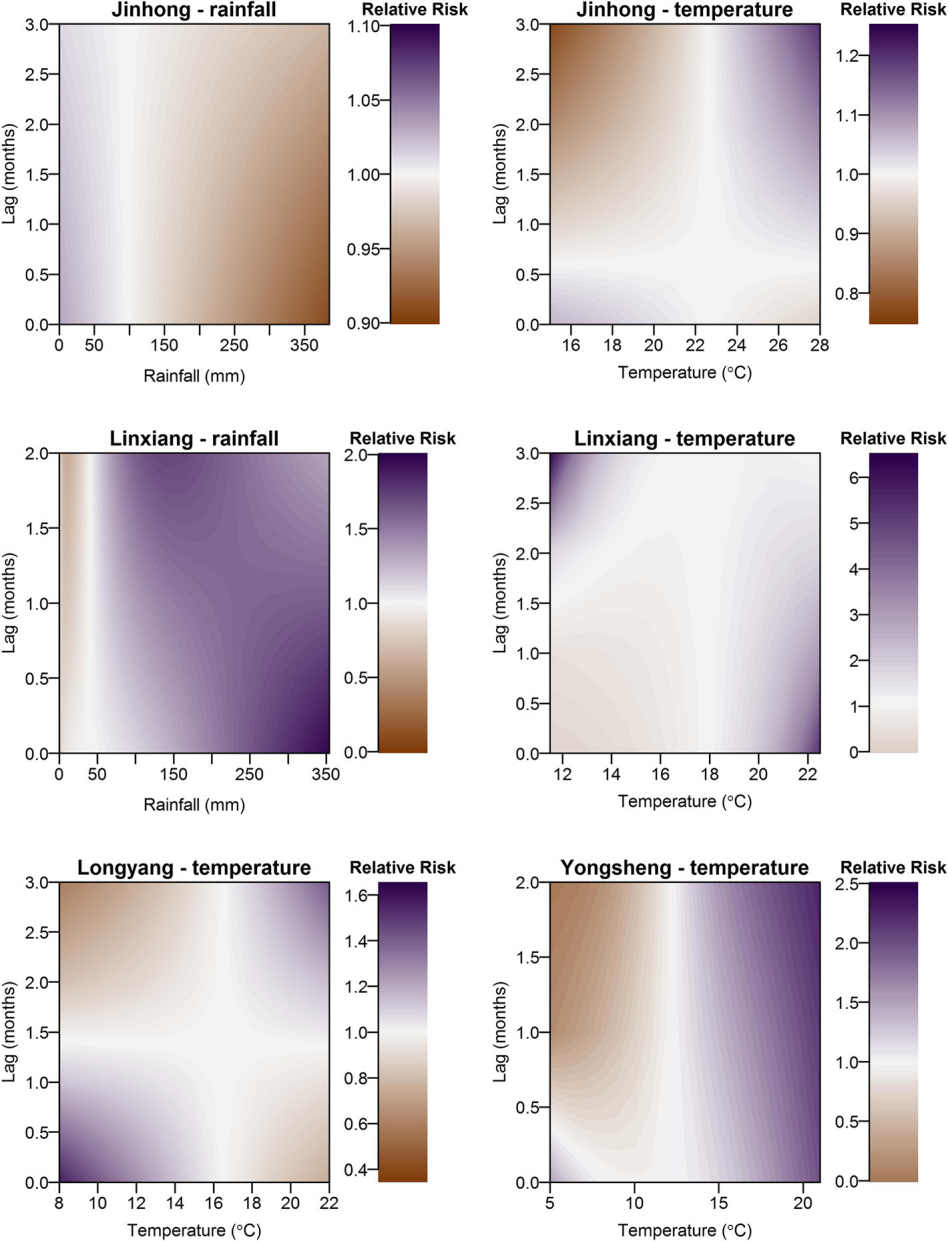
**Filled contour plot illustrating the lagged effects of temperature and rainfall on incidence of malaria for the four counties.** High relative risks (> 1) are indicated by purple shading and low relative risks (<1) are indicated by brown shading.

The best regression model for Jinhong included rainfall and temperature, both at maximum lags of three months (see Figure 8, top panels for contour plots of the predicted relative risk by rainfall, temperature and lag). RRs smaller than one occurred following high rainfall (>100 mm) at lags of zero to three months. RRs greater than one were predicted at high temperatures (>22°C) for lags of one to three months, and RRs less than one at low temperatures (<22°C) for the same lag periods.

For Linxiang, the final model included logged rainfall with a maximum lag of two months, and temperature with a maximum lag of three months (Figure 8, central panels). High rainfall (>50 mm) at lags of zero to two months resulted in RRs greater than one, whereas low rainfall (< 25 mm) at these lags resulted in RRs of less than one. Months with low temperatures (<18°C) in Linxiang had RRs of less than one, whereas temperatures of over 19°C at lags of zero to three months resulted in high RRs.

The final model for Longyang included temperature at a maximum lag of three months (Figure 8, bottom left panel). High temperature (>17°C) at lags of two to three months and low temperature (<17°C) with no lag resulted in RRs greater than one. The final model for Yongsheng (Figure 8, bottom right panel) contained the log of temperature at a maximum lag of two months. Temperatures greater than 14°C at a lag of zero to two months resulted in RRs greater than one, whereas low temperature (<12°C) resulted in RRs of less than one.

## Discussion

The use of time-series analysis can provide valuable insights into the seasonal patterns of malaria transmission, and the influence of the weather on these patterns. Thorough understanding of malaria seasonality will help the adaptation of malaria control and elimination measures, for example via the targeted timing of bed-net distribution or indoor residual spraying to ensure maximum efficacy. The analysis presented here highlights the heterogeneity in seasonal patterns in malaria in four counties within the same province of China, driven by weather, potentially in combination with local ecology, biological and socio-economic factors.

In all four counties a declining secular trend was noted, which mirrors that seen in other parts of China [5,7,8] and globally [21]. This is likely to have resulted from socio-economic development (e.g., improved housing, increased access to health care) and the implementation of malaria control activities. Yunnan province has benefitted from national malaria control initiatives, funding from the Global Fund to Fight AIDS Tuberculosis and Malaria (2003–2011), the Mekong Roll Back Malaria Information, Education and Communication project (2002–2004) and the Strengthening Malaria Control for Ethnic Minorities project (2006–2007) [6]. However, the declining incidence of malaria was detected prior to the initiation of these large projects, indicating the role of socio-economic improvements within Yunnan province.

The two distinct patterns over time in the residuals from the decomposition analysis suggest that the pattern of malaria transmission altered around 1998 or 1999. The smaller residual values after this transition may, in part, be due to the overall decreasing trend in the data. However, this would not explain the different temporal patterns. Other potential explanations would be the initiation of a specific control programme at that time (that could have altered the natural transmission dynamics of malaria), changes in cross-border movement from neighbouring countries, or changes in agricultural policies (e g., irrigation practices).

The use of lagged non-linear models allowed an assessment of the delayed effects of temperature and rainfall on malaria incidence. Published research generally agrees that rainfall and temperature are important drivers of seasonal patterns of malaria, although the precise relationships, including the most significant lag periods, vary between different settings, and temporal models developed cannot be generalized across different areas [12,22].

Lagged temperature was a significant variable in the final models for all four counties (three month lag for Jinhong, Linxiang and Longyang and two month lag for Yongsheng), with increased temperatures relating to increased risk. The exact relationships varied between counties in terms of the temperature threshold for increased risk, and the effect at shorter (1 month) lags. In general, temperatures below 16°C are detrimental for mosquito survival and *P. vivax* parasite development, thus, transmission risk below this temperature is negligible [23,24]. In warmer climates, where temperature is less of a constraint on malaria transmission (as is likely the case in Jinhong), increasing temperature results in shorter mosquito development periods, shorter parasite development periods and increased biting behaviour by the mosquitoes, thus, producing increased malaria transmission [12,13]. The relationships detected correspond well with this biological understanding of mosquito and *Plasmodium* development in relation to temperature and the differences in the specific relationships for each county may be accounted for by differences in the climatic profile of each area.

Temperature was the main constraining factor for malaria incidence in both Yongsheng and Longyang, whereas rainfall was also included in the final models for Linxiang and Jinhong. The relationships between rainfall and malaria incidence varied between these two counties, with increased rainfall associated with lower incidence of malaria in Jinhong (up to a lag of three months) and increased incidence of malaria in Linxiang (up to a lag of two months). Previous studies have indicated increased risk following high rainfall [5,25-28], which may be expected due to the dependency of mosquito replication and survival on water [10]. However, other studies have detected the opposite (as was observed in Jinhong), or no association [11,28,29]. A potential explanation for decreased risk following high rainfall is that large precipitation volumes may wash away mosquito larvae, thus, decreasing vector populations and malaria risk [11,12], although further factors such as land cover (or use), soil and hydrology may contribute to the lack of consistency in observed relationships across different study areas. The results from the distributed lag non-linear models add further understanding of the temporal epidemiology of malaria in Yunnan province and the lagged effects of weather variables on disease incidence.

Imported malaria cases from bordering countries, particularly Myanmar, play an important role in the temporal epidemiology of malaria in Yunnan province, with seasonal patterns of imported cases correlated with overall malaria seasonality in China [30]. Jinhong shares a border with Myanmar, while the other three counties are between within 250 km from the border, suggesting that imported malaria cases and, thus, climatological conditions in neighbouring Myanmar may also be important drivers for seasonal patterns in malaria in Yunnan province. Additional agricultural and socio-economic factors, such as irrigation and human behaviour are intrinsically linked to malaria risk: seasonal changes in these factors may have important implications for malaria transmission, although it was not possible to include these factors in this analysis.

Despite the significance of weather variables in seasonal patterns of malaria, this analysis was not able to provide any evidence of a link between weather anomalies and unusually high incidence of malaria (e g, unseasonably high rainfall leading to a peak in malaria incidence), which is in line with previous findings [12,31]. This may indicate that unusually high incidence of malaria is not driven by unusual weather patterns in the counties, and rather, is mediated by other factors such as human behaviour, movement patterns, migration, or agricultural practices. However, the analysis presented did not consider interactions: examining combinations of weather variables may reveal a more complex scenario.

## Conclusion

The information provided here may allow the temporal targeting of malaria elimination efforts at a local level within Yunnan province. Implementation of malaria interventions during February (or January in Longyang) may improve their efficacy by reducing mosquito populations (e g, via residual insecticides) and biting rates (eg, via ITN delivery) from the start of the malaria season, prior to increasing malaria transmission. The results presented here illustrate the complex relationships between rainfall, temperature and malaria incidence and highlight the important differences in these relationships in heterogeneous landscapes and climates. Local knowledge of seasonal weather patterns and how these influence malaria transmission, in combination with an understanding of the local vector species, is vital for effective malaria control.

## Competing interests

The authors declare that they have no competing interests.

## Authors’ contributions

NAW carried out statistical analysis and interpretation of results, and wrote the manuscript. AGB contributed to statistical analysis, interpretation and editing of the manuscript. J-AA carried out preliminary statistical analysis and contributed to interpretation and editing of the manuscript. ACAC conceived of the study, acquired and formatted the data and contributed to statistical analysis, interpretation and editing of the manuscript. All authors read and approved the final manuscript.

## Supplementary Material

Additional file 1: Table S1 Correlation between atypical malaria incidence (residuals from the seasonal trend decomposition) and atypical rainfall and temperature (residuals from the linear regression accounting for seasonality and trend, where appropriate) for each of the four counties over lags of zero to six months. Click here for file

Additional file 2: Table S2 Parameter estimates from final models for Jinhong. Click here for file

Additional file 3: Table S3 Parameter estimates from final models for Longyang. Click here for file

Additional file 4: Table S4 Parameter estimates from final models for Yongsheng. Click here for file

Additional file 5: Table S5 Parameter estimates from final models for Linxiang. Click here for file

Additional file 6: Figure S1 Temporal trend for the four counties. Click here for file
