# Supplementary material for: Plasmodium vivax malaria incidence over time and its association with temperature and rainfall in four counties of Yunnan Province, China
Source: Malar J. 2013 Dec 18;12:452. doi: 10.1186/1475-2875-12-452 (PMC3878361; doi:10.1186/1475-2875-12-452)
Supplement: Additional file 1: Table S1 — Correlation between atypical malaria incidence (residuals from the seasonal trend decomposition) and atypical rainfall and temperature (residuals from the linear regression accounting for seasonality and trend, where appropriate) for each of the four counties over lags of zero to six months. [file 1475-2875-12-452-S1.pdf]

Table S1 Correlation between atypical malaria incidence (residuals from the seasonal trend decomposition) and atypical rainfall and temperature (residuals from the linear regression accounting for seasonality and trend, where appropriate) for each of the four counties over lags of zero to six months.

	**Jinhong**		**Linxiang**		**Longyang**		**Yongsheng**	
**Lag**	**Rain**	**Temp**	**Rain**	**Temp**	**Rain**	**Temp**	**Rain**	**Temp**
**0**	-0.05	0.07	-0.04	0.004	0.0005	-0.04	0.02	0.09
**1**	-0.13	0.09	-0.17	0.07	0.04	-0.01	0.09	0.12
**2**	-0.16	0.09	-0.14	0.05	-0.01	-0.01	0.04	0.14
**3**	-0.19	0.17	-0.12	0.05	0.006	0.03	0.06	0.17
**4**	-0.20	0.14	-0.14	0.12	-0.01	-0.004	0.05	0.21
**5**	-0.18	0.14	-0.13	0.14	-0.06	0.05	0.05	0.22
**6**	-0.16	0.16	-0.13	0.11	-0.08	0.04	0.05	0.21
