# Supplementary material for: Plasmodium vivax malaria incidence over time and its association with temperature and rainfall in four counties of Yunnan Province, China
Source: Malar J. 2013 Dec 18;12:452. doi: 10.1186/1475-2875-12-452 (PMC3878361; doi:10.1186/1475-2875-12-452)
Supplement: Additional file 2: Table S2 — Parameter estimates from final models for Jinhong. [file 1475-2875-12-452-S2.pdf]

Table S2: Parameter estimates from final model for Jinhong.

	Estimate	*p* - value	Risk Ratio	2.50%	97.50%
**Intercept**	-10.85	<0.005	1.95×10^{-5}	1.36×10^{-5}	2.78×10^{-5}
**Trend[1]**	-0.75	<0.005	0.47	0.40	0.56
**Trend[2]**	-0.71	<0.005	0.49	0.45	0.53
**Feb**	0.78	<0.005	2.17	1.67	2.84
**Mar**	0.95	<0.005	2.59	1.80	3.75
**Apr**	1.18	<0.005	3.24	2.05	5.14
**May**	1.49	<0.005	4.45	2.68	7.40
**Jun**	2.31	<0.005	10.03	5.87	17.21
**Jul**	2.33	<0.005	10.31	6.00	17.78
**Aug**	2.56	<0.005	12.99	7.56	22.41
**Sep**	2.55	<0.005	12.76	7.63	21.41
**Oct**	1.49	<0.005	4.47	2.82	7.03
**Nov**	0.73	<0.005	2.08	1.47	2.95
**Dec**	-0.46	<0.005	0.63	0.47	0.85
**Rainfall[a1]**	-0.22	0.01	0.80	0.67	0.96
**Rainfall[a2]**	0.06	0.53	1.06	0.86	1.30
**Temperature[a1]**	0.36	0.17	1.43	0.86	2.37
**Temperature[a2]**	0.52	0.07	1.68	0.97	2.92

[a]Natural splines with 2 *df* were used for the trend and the lag effects.
