# Supplementary material for: Plasmodium vivax malaria incidence over time and its association with temperature and rainfall in four counties of Yunnan Province, China
Source: Malar J. 2013 Dec 18;12:452. doi: 10.1186/1475-2875-12-452 (PMC3878361; doi:10.1186/1475-2875-12-452)
Supplement: Additional file 3: Table S3 — Parameter estimates from final models for Longyang. [file 1475-2875-12-452-S3.pdf]

Table S3: Parameter estimates from final model for Longyang.

	Estimate	*p* - value	Risk Ratio	2.50%	97.50%
**Intercept**	-13.07	<0.005	2.11×10^{-6}	1.02×10^{-6}	4.32×10^{-6}
**Trend**	-1.21	<0.005	0.30	0.23	0.38
**Feb**	1.94	<0.005	6.95	4.20	11.57
**Mar**	1.94	<0.005	6.96	3.04	16.01
**Apr**	0.31	0.58	1.36	0.46	3.97
**May**	1.44	0.01	4.22	1.40	12.71
**Jun**	1.82	0.002	6.18	1.94	19.79
**Jul**	2.54	<0.005	12.63	3.97	40.33
**Aug**	2.37	<0.005	10.65	3.42	33.37
**Sep**	2.00	<0.005	7.41	2.56	21.62
**Oct**	0.67	0.17	1.96	0.76	5.08
**Nov**	0.36	0.32	1.43	0.71	2.90
**Dec**	-0.48	0.08	0.62	0.36	1.06
**Temperature[a1]**	0.06	0.91	1.06	0.39	2.87
**Temperature[a2]**	1.58	0.02	4.88	1.24	19.19

[a]Natural splines with 2 *df* were used for the lag effects.
