# Supplementary material for: Plasmodium vivax malaria incidence over time and its association with temperature and rainfall in four counties of Yunnan Province, China
Source: Malar J. 2013 Dec 18;12:452. doi: 10.1186/1475-2875-12-452 (PMC3878361; doi:10.1186/1475-2875-12-452)
Supplement: Additional file 4: Table S4 — Parameter estimates from final models for Yongsheng. [file 1475-2875-12-452-S4.pdf]

Table S4: Parameter estimates from final model for Yongsheng.

	**Estimate**	***p* - value**	**Risk Ratio**	**2.50%**	**97.50%**
**Intercept**	12.11	<0.005	5.50×10^{-6}	4.18×10^{-6}	7.19×10^{-6}
**Trend**	-1.85	<0.005	0.16	0.12	0.21
**spring**	0.61	<0.005	1.85	1.25	2.71
**summer**	0.33	0.008	1.39	1.09	1.77
**winter**	-0.94	0.04	0.39	0.16	0.94
**Temperature[a1.1]**	6.96	<0.005	1.058×10^{3}	65.94	2.15×10^{04}
**Temperature[a2.1]**	2.95	<0.005	19.07	11.31	33.55
**Temperature[a1.2]**	7.21	<0.005	1.36×10^{3}	97.84	2.12×10^{04}
**Temperature[a2.2]**	1.61	<0.005	5.02	2.68	9.52

[a] Natural splines with 2 *df* were used for the lag and covariate effects.
