# Supplementary material for: Plasmodium vivax malaria incidence over time and its association with temperature and rainfall in four counties of Yunnan Province, China
Source: Malar J. 2013 Dec 18;12:452. doi: 10.1186/1475-2875-12-452 (PMC3878361; doi:10.1186/1475-2875-12-452)
Supplement: Additional file 5: Table S5 — Parameter estimates from final models for Linxiang. [file 1475-2875-12-452-S5.pdf]

Table S5: Parameter estimates from final model for Linxiang.

	Estimate	*p* - value	Risk Ratio	2.50%	97.50%
**Intercept**	-13.88	<0.005	9.38×10^{-7}	5.22×10^{-7}	1.62×10^{-6}
**Trend[1]**	-0.50	0.10	0.61	0.34	1.10
**Trend[2]**	-1.04	<0.005	0.35	0.25	0.49
**Rainfall[a1.1]**	0.97	0.09	2.65	0.89	8.53
**Rainfall[a2.1]**	0.04	0.98	1.04	0.06	22.45
**Rainfall[a3.1]**	1.60	<0.005	4.94	2.43	10.30
**Rainfall[a1.2]**	0.49	0.27	1.63	0.67	3.79
**Rainfall[a2.2]**	-1.56	0.14	0.21	0.02	1.60
**Rainfall[a3.2]**	0.15	0.64	1.16	0.62	2.14
**Temperature[a1.1]**	1.60	0.01	4.95	1.53	20.00
**Temperature[a2.1]**	1.61	0.43	4.98	0.13	341.21
**Temperature[a3.1]**	2.91	<0.005	18.34	9.88	36.40
**Temperature[a1.2]**	-3.36	<0.005	0.03	0.01	0.10
**Temperature[a2.2]**	-8.82	<0.005	1.48×10^{-4}	4.91×10^{-6}	2.48×10^{-3}
**Temperature[a3.2]**	-3.08	<0.005	0.05	0.02	0.08

[a]A 3 *df* natural spline was included on the covariate effects and a 2 *df* natural spline on the lag effects and the trend.
