# Supplementary material for: Plasmodium vivax malaria incidence over time and its association with temperature and rainfall in four counties of Yunnan Province, China
Source: Malar J. 2013 Dec 18;12:452. doi: 10.1186/1475-2875-12-452 (PMC3878361; doi:10.1186/1475-2875-12-452)
Supplement: Additional file 6: Figure S1 — Temporal trend for the four counties. [file 1475-2875-12-452-S6.pdf]

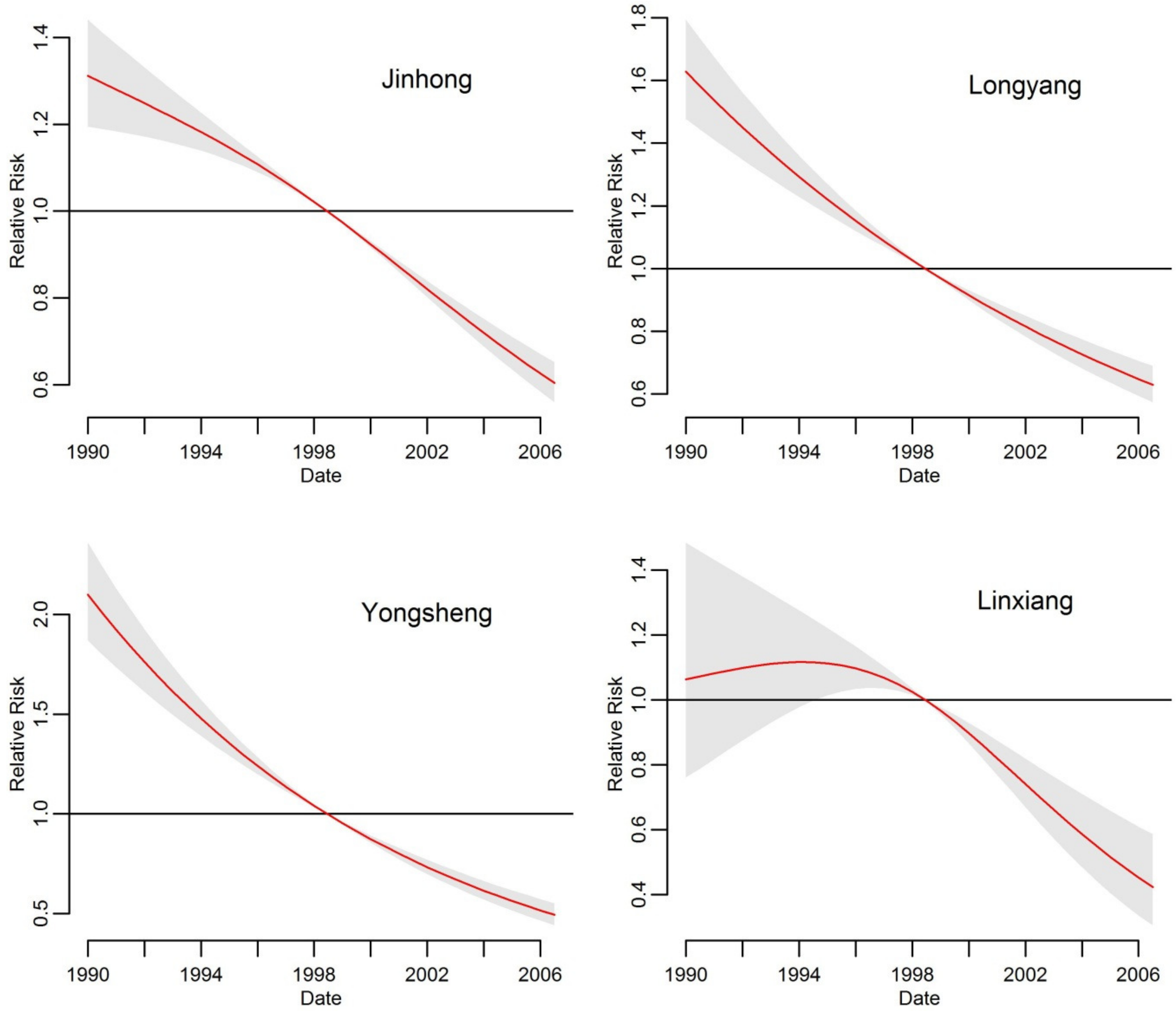


**Figure s1: Temporal trend for Jinhong (top left), Longyang (top right), Yongsheng (bottom left) and Linxiang (bottom right).**
